# Genomic Analysis of Bacteriocin-Producing Staphylococci: High Prevalence of Lanthipeptides and the Micrococcin P1 Biosynthetic Gene Clusters

**DOI:** 10.1007/s12602-023-10119-w

**Published:** 2023-08-26

**Authors:** Rosa Fernández-Fernández, Ahmed M. A. Elsherbini, Carmen Lozano, Agustí Martínez, María de Toro, Myriam Zarazaga, Andreas Peschel, Bernhard Krismer, Carmen Torres

**Affiliations:** 1 Area of Biochemistry and Molecular Biology, OneHealth-UR Research Group, University of La Rioja, 26006 Logroño, Spain; 2 Department of Infection Biology, Interfaculty Institute of Microbiology and Infection Medicine, University of Tübingen, 72076 Tübingen, Germany; 3 Cluster of Excellence EXC 2124 Controlling Microbes to Fight Infections, Tübingen, Germany; 4 German Center for Infection Research (DZIF), Partner Site Tübingen, Tübingen, Germany; 5 Genomics and Bioinformatics Core Facility, Center for Biomedical Research of La Rioja, Logroño, Spain

**Keywords:** Bacteriocins, BGC, CoNS, Lanthipeptides, Micrococcin P1

## Abstract

Bacteriocins are antimicrobial peptides produced by bacteria. This study aimed to in silico analyze the presence of bacteriocin gene clusters (BGCs) among the genomes of 22 commensal *Staphylococcus* isolates from different origins (environment/human/food/pet/wild animals) previously identified as bacteriocin producers. The resistome and plasmidome were studied in all isolates. Five types of BGC were detected in 18 genomes of the 22 bacteriocin-producing staphylococci included in this study: class I (Lanthipeptides), class II, circular bacteriocins, the non-ribosomal-peptide lugdunin and the thiopeptide micrococcin P1 (MP1). A high frequency of lanthipeptides was detected in this collection: BGC variants of BSA, bacCH91, and epilancin15X were identified in two *Staphylococcus aureus* and one *Staphylococcus warneri* isolates from food and wild animals. Moreover, two potentially new lanthipeptide-like BGCs with no identity to database entries were found in *Staphylococcus epidermidis* and *Staphylococcus simulans* from food and wild animal, respectively. Interestingly, four isolates (one *S. aureus* and one *Staphylococcus hominis*, environmental origin; two *Staphylococcus sciuri*, food) carried the MP1 BGC with differences to those previously described. On the other hand, seven of the 22 genomes (~32%) lacked known genes related with antibiotic or disinfectant-acquired resistance mechanisms. Moreover, the potential carriage of plasmids was evaluated, and several Rep-proteins were identified (~73% of strains). In conclusion, a wide variety of BGCs has been observed among the 22 genomes, and an interesting relationship between related *Staphylococcus* species and the type of bacteriocin has been revealed. Therefore, bacteriocin-producing *Staphylococcus* and especially coagulase-negative staphylococci (CoNS) can be considered good candidates as a source of novel bacteriocins.

**Supplementary Information:**

The online version contains supplementary material available at 10.1007/s12602-023-10119-w.

## Introduction

Antimicrobial resistance (AMR) represents one of the biggest challenges of modern medicine worldwide [1]. This AMR global problem requires novel antimicrobial agents and strategies to overcome the threat by various pathogens, including multidrug-resistant (MDR) and zoonotic bacteria [2, 3]. About 35% of all drugs and 60–80% of all antimicrobial products have originated from natural products, and the development of innovative products derived from natural substances is gaining attention for pharmaceutical and therapeutic uses [4–6]. However, financial pressure from drug companies as well as difficulties in the isolation and identification of new natural compounds have severely limited the discovery rate from these important sources [5, 6].

Typically, bacteriocins are ribosomally synthesized peptides, encoded by operons including the structural genes whose products are often post-translationally modified by specific enzymes encoded by adjacent genes. In these genetic systems, regulatory genes, immunity genes, transporters, and other genes that encode accessory proteins are also found [7–9]. Traditionally, the identification of novel bacteriocins has used cultivation-based approaches that involved screening of numerous isolates for antimicrobial activity, followed by long-term biochemical characterization [10, 11]. More recently, genome mining has become an important methodology in the discovery of novel natural products with antimicrobial activity [7]. In this respect, ribosomally synthesized and post-translationally modified peptides (RiPPs) of bacterial origin are natural compounds that are highly attractive candidates for antibacterial prevention and therapy [7, 12]. Nevertheless, other types of bacteriocins can be also very relevant such as Aureocins or Epidermicin NI01 [13].

Bacteriocins have been divided in three major classes depending on their amino acid composition, chemical structure, complexity, mode of action, and steps involved in their production (synthesis, transport, and immunity): class I, small post-translationally modified peptides; class II, unmodified bacteriocins; and class III, larger and thermo-labile peptides [14, 15]. Moreover, recent reviews have proposed the inclusion of additional classes among Gram-positive bacteriocins: class IV (circular), class V (sactipeptides), class VI (thiopeptides) and non-ribosomal peptides (NRP) [13, 14, 16–22].

Within the last few years, increasing numbers of bacteriocins have been isolated and identified from Gram-positive microorganisms [15], and the lanthipeptides (class I) belong to the most frequently found [23]. These peptides represent a promising type of natural antibacterial molecules, active against many Gram-positive pathogens, including antibiotic-resistant strains such as methicillin-resistant *S. aureus* (MRSA) and vancomycin-resistant enterococci (VRE) [24].

Environmental members of the phylum Actinobacteria and Firmicutes including bacilli and lactic acid bacteria, and many Proteobacteria such as* Escherichia coli* are well-known as producers of biologically active substances [25–27]. In addition, *Staphylococcus* species, in particular coagulase-negative staphylococci (CoNS) isolated from a wide variety of natural sources, have also been found to be interesting sources of BGCs for bacteriocins [28–30].

In previous studies carried out by our group [31–33], a collection of 92 bacteriocin-producing staphylococci was detected in a screening approach performed with 1205 staphylococci of different species and origins (~7.7% were bacteriocin producers) (Table S1). Twenty-two of these isolates of 11 species, both coagulase-positive and negative staphylococci (CoPS and CoNS, respectively), were included in this study for further characterization. Isolates were selected under a OneHealth perspective based on their antimicrobial activity profile (high production) and their origin. The specific objectives of this study were (1) to predict and analyze the presence of BGCs in the genomes of the 22 selected bacteriocin-producing *Staphylococcus* isolates and (2) to investigate the novelty or the homology between our BGCs after comparison with those previously reported in databases.

## Material and Methods

### Isolates Included in the Study

The genomes of 22 *Staphylococcus* isolates, both CoPS and CoNS, of eleven different species and six origins, were included in this study for genomic comparison. The bacteriocin-producing isolates were as follows (number of isolates, identification code): (a) *S. aureus* from food (*n* = 1, X3410), environment (*n* = 1, C5802), and wild mammal (*n* = 1, C8609); (b) *Staphylococcus pseudintermedius* from dogs (*n* = 2, C4502, C8478) and human (*n* = 1, C8189); (c) *Staphylococcus chromogenes* from wild mammals (*n* = 2, C9838, C9727); (d) *Staphylococcus hyicus* isolates from wild mammals (*n* = 2, C9581, C9585); (e) *S. sciuri* isolates recently reclassified as *Mammaliicoccus sciuri* [34, 35] were recovered from wild birds (*n* = 3, C9213, C9179, C9529) and food (*n* = 2, X3011, X3041); (f) *Staphylococcus lugdunensis* isolates from humans (*n* = 2, C9161, C9954); (g) *S. hominis* of environmental origin (*n* = 1, C5835); (h) *S. warneri* (*n* = 1, X2969) from food; (i) *S. simulans* recovered from a wild mammal (*n* = 1, C9832); (j) *S. epidermidis* (*n* = 1, X3009), from food and (k) *Staphylococcus xylosus* (*n* = 1, C9255) isolated from wild birds. The characteristics of the 22 isolates analyzed in this study are indicated in Table S2.

### DNA Extraction, Amplification, and Sequencing

The NucleoSpin microbial DNA Kit (Macherey and Nagel, Germany) was used for DNA extraction. For it, 2 ml of a 24-h grown culture in normal BM medium at 37 °C were pelleted (40 mg) by centrifugation (10 min at 10,000 rpm), and the FastPrep-24 Classic homogenizer (MP Biomedicals) was used for mechanical disruption. Genomic DNA was subsequently purified according to the manufacturers’ instructions.

The genomic libraries for eight *Staphylococcus* isolates (X2969, X3009, X3041, C8609, C9832, C9581, C9585, and C9838) were prepared with the TruSeq DNA PCR-Free Kit (Illumina) by the Genomics and Bioinformatics Core Facility (CIBIR), whereas the genomic libraries of the other isolates (C4502, C9161, C9179, C9213, C9255, C9529, C9727, C9954, X3011, and X3410) were prepared with the Nextera XT library preparation kit (Illumina) by the NGS Competence Center Tübingen (NCCT). In both cases, libraries were sequenced in different runs on an Illumina MiSeq platform with the MiSeq Reagent Kit v3 (Illumina, 150 cycle). The genomes of the isolates C5802, C5835, C8478, and C8189 were previously obtained in collaborative studies of the OneHealth-UR research group and all isolates belong to the strain collection of the University of La Rioja.

The Whole Genome project has been deposited at GenBank under the accession PRJNA974190 and the respective accession numbers are included in Table S2.

### Data Analysis

Raw reads quality was checked using FastQC (v0.11.8) [36]. Trimming of raw reads, de novo assembly, and assembly polishing were performed with Trimmomatic (v0.39) [37], SPAdes (v3.15.5), and Pilon (v1.24), respectively [38, 39], through the Shovill pipeline [40]. Then, assembled genomes were functionally annotated using Prokka (v1.14.6) [41].

Using protein files exported from the Prokka annotations in FASTA format, a maximum likelihood tree was computed via the RaxML program (v8.2.12) [42], applied inside PhyloPhlAn (v3.0.60) pipeline using the PhyloPhlAn protein markers database and with the default diversity parameter for genus/family level phylogenies [43]. The tree was visualized using the ggtree package (v3.4.4) of R (v4.2.1) [44, 45].

### Resistome and Plasmidome

The resistance genotypes and the presence of plasmid replication (*rep*) genes were studied through ResFinder and PlasmidFinder web tools, respectively [46]. In all cases, the default parameters with relaxed mode were used and *S. aureus* was selected as reference species.

### Bacteriocin Gene Clusters Prediction

To predict and analyze homologous bacteriocin gene clusters (BGCs) in the 22 genomes, all annotated genomes in GenBank format were uploaded to antiSMASH (v6.1.1) [47] and BAGEL4 [48] with all parameters and a relaxed mode. For BGC prediction, the genomes were considered as a pool of contigs both including chromosome and plasmid regions.

To extract homologous BGCs of micrococcin P1 (MP1) from *Macrococcus caseolyticus*, pBac115 plasmid sequence (accession number KM613043.1) was used as reference, and the protein sequences identified by antiSMASH were blasted against the NCBI protein database using the Cblaster (v1.3.15) tool with default parameters [49]. To simplify the output, unique non-redundant homologous BGCs per species were aligned against the *M. caseolyticus* BGC and the MP1 genomes from this study using the Clinker (v0.0.25) tool with the default parameters [50].

To investigate whether all identified micrococcin P1 BGCs were encoding the identical thiopeptide, the structural protein sequences were extracted from all BGCs of interest. Then, they were mutually aligned by MAFFT (v7.310) and visualized with Jalview (v2.11.2.0) according to ClustalX color scheme [51].

Moreover, identity comparisons between the MP1 plasmid from *S. hominis* S34-1 (CP040733.1) and the MP1-producing isolates (*S. sciuri* X3041 and X3011, *S. hominis* C5835, *S. aureus* C5802) were performed with Clustal W2 and Emboss Needle [52], and Clinker (v0.0.25) tools [50].

## Results

The complete genomes of 22 bacteriocin-producing *Staphylococcus* spp. isolates, both CoPS and CoNS were obtained by WGS and annotation. Phenotypic and molecular characteristics of the isolates as well as results of resistome and plasmidome analyses are presented in Tables S2 and S3. Several bacteriocin-encoding gene clusters were identified, which are summarized in Table 1.

**Table 1:** Details of the type of bacteriocin gene clusters (BGC) predicted both by antiSMASH or BAGEL4 among the 22 genomes included in this study

**Isolate**	**Specie**	**Origin**	**BGC type(s)**^**a**^	**BGC prediction**^**a,b,c**^
X3041	*S. sciuri*	Food	Class II, thiopeptide	Lactococcin972, MP1
X3011	*S. sciuri*	Food	Class II, thiopeptide	Lactococcin972, MP1
C9179	*S. sciuri*	Wild bird	NRPs	None^c^
C9213	*S. sciuri*	Wild bird	NRPs	None^c^
C9529	*S. sciuri*	Wild bird	NRPs	None^c^
C9585	*S. hyicus*	Wild mammal	Lanthipeptide	Lanthipeptide V
C9581	*S. hyicus*	Wild mammal	RiPP	Circular
C9838	*S. chromogenes*	Wild mammal	RiPP	2 Circular
C9727	*S. chromogenes*	Wild mammal	Lanthipeptide	Hominicin variant
C9832	*S. simulans*	Wild mammal	Class II, lanthipeptide	Lactococcin972, new lanthipeptide
C5835	*S. hominis*	Environmental	Thiopeptide	MP1
C9255	*S. xylosus*	Wild bird	Class II, RiPP	Lactococcin972, blp, circular
X2969	*S. warneri*	Food	Lanthipeptide	Epilancin15X variant
X3009	*S. epidermidis*	Food	Lanthipeptide	New lanthipeptide
C8478	*S. pseudintermedius*	Pet	Class II	Bacsp222
C8189	*S. pseudintermedius*	Human	Class II	Bacsp222
C4502	*S. pseudintermedius*	Pet	NRPs	None^c^
C5802	*S. aureus*	Environmental	Class II, thiopeptide	Lactococcin972, MP1
C8609	*S. aureus*	Wild mammal	Class II, lanthipeptide	Lactotococcin972, BSA
X3410	*S. aureus*	Food	Lanthipeptide	BacCH91
C9954	*S. lugdunensis*	Human	NRPs	Lugdunin
C9161	*S. lugdunensis*	Human	NRPs	Lugdunin

^a^Abbreviation: *MP1* micrococcin P1, *NRPs* non-ribosomal-peptide-synthetase, *RiPP* ribosomally synthesized and post-translationally modified peptide

^b^Bacteriocin names: bacsp222, BSA, bacCH91

^c^None: in these cases, the relation of the predicted secondary metabolites with antimicrobial substances could not be verified

According to the analysis of the 22 genomes included in this study, seven out of 22 (~32%) isolates lacked of known antibiotic or disinfectant resistance-related genes. The remaining 15 isolates (~68%) carried at least one gene associated with antibiotic resistance (beta-lactams, macrolides, lincosamides, streptogramins and aminoglycosides (LSA), tetracyclines, phenicol, fusidic acid, and fosfomycin) or disinfectant agents. Regarding the plasmidome, a wide variety of Rep protein genes were identified in 16 of the studied genomes. Among these genomes, eight carried *rep* and antimicrobial resistance genes and the other eight only carried *rep* genes (Table S2). Interestingly, isolates C9727, C9954, and C9161 did not carry any potential antimicrobial resistance gene, disinfectant-related resistance mechanism or plasmids (lack of Rep protein genes) (Tables S2 and S3).

**Fig. 1:**
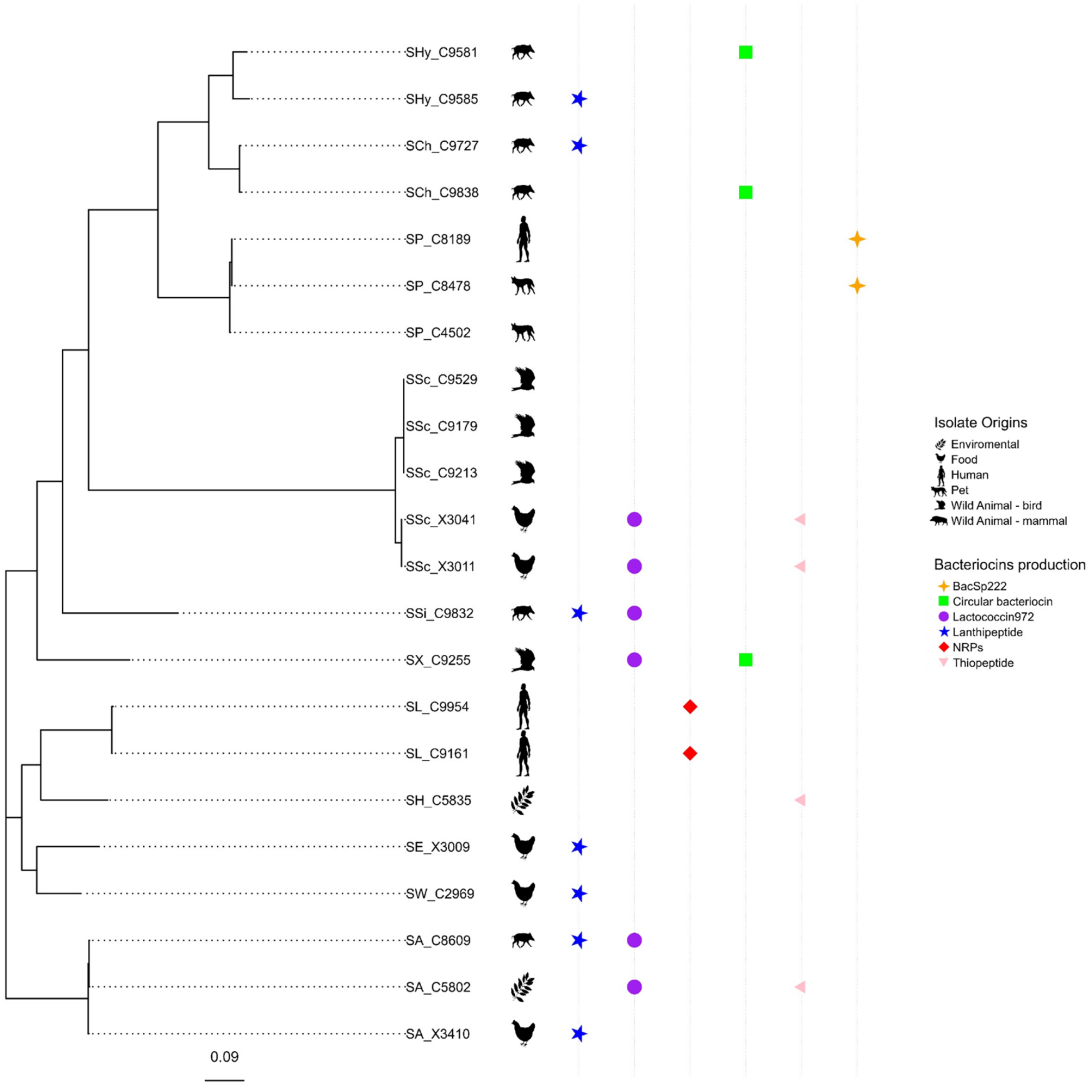
Maximum likelihood tree of protein markers of the 22 bacteriocin-producing isolates of different origins (environmental, food, human, pet, and wild animal both birds and mammals). The bacteriocin gene clusters (BGC) identified after in silico analysis are marked with symbols and colors: class I, lanthipeptides (blue star); class II, lactotoccin972 (purple circle) and bacsp222 (yellow cross); circular (green square), NRPS (red rhombus), thiopeptide (pink triangle). Abbreviation of specie: SA *S. aureus*, SP *S. pseudintermedius*, SSc *S. sciuri*, SCh *S. chromogenes*, SSi *S. simulans*, SX *S. xylosus*, SL *S. lugdunensis*, SH *S. hominis*, SE *S. epidermidis*, SW *S. warneri*. This figure is in color in the online version but in black and white in the print version

The use of antiSMASH and BAGEL4 software revealed the presence of a large number and wide diversity of BGCs in the various genomes (Table 1). Comparison of all predicted BGCs identified five types of BGCs in 18 of the 22 genomes of bacteriocin-producing isolates from different species and origins included in this study (Fig. 1). The BGCs from this study were assigned to class I (lanthipeptides), class II (bacSp222, lactococcin972 and blp), circular bacteriocins, NRPs (lugdunin), and thiopeptide (MP1), as follows:

1. Class I: Seven out of the 22 genomes (~32%) carried a lanthipeptide-like BGCs with identities to database entries. The isolates were recovered from wild animals (*S. aureus* C8609 and *S. simulans* C9832) or food (*S. aureus* X3410, *S. warneri* X2969, *S. epidermidis* X3009). Interestingly, two of these isolates (C9832 and X3009) seemed to carry currently unknown lanthipeptide-encoding BGCs. Moreover, a type V lanthipeptide was detected in *S. hyicus* isolate C9585 recovered from a wild animal (wild boar), and strong identity was found between the BGC predicted in *S. chromogenes* C9727 (wild boar) and the structural gene coding for the lanthipeptide hominicin.
2. Class II: Genes for lactococcin972-like peptides were detected among 6 out of 22 genomes (~36%) from (a) three isolates recovered from wild animals (*S. aureus* C8609, *S. xylosus* C9255 and *S. simulans* C9832); (b) two *S. sciuri* isolates from food samples (X3041 and X3011); and (c) one *S. aureus* C5802 isolate recovered from an environmental sample. Moreover, part of the bacSp222 bacteriocin gene cluster was detected in two *S. pseudintermedius* (C8478 and C8189) isolates recovered from the same dog–human zoonosis case and a *blp*-like bacteriocin operon was identified in *S. xylosus* C9255.
3. BGCs for circular bacteriocins were identified in three isolates recovered from wild animals (*S. chromogenes* C9838, *S. hyicus* C9581, and *S. xylosus* C9255). The predicted BGCs revealed high identity with uberolysin/circularinA and aureocyclicin 4185 described in the databases for other species.
4. Non-ribosomal-peptide-synthetase genes (NRPS) coding for lugdunin were detected in two *S. lugdunensis* isolates (C9161 and C9954) recovered from humans.
5. Four isolates (~18%) carried a BGC coding for the thiopeptide micrococcin P1: *S. aureus* C5802 and *S. hominis* C5835 from environmental samples and two *S. sciuri* X3011 and X3041 from food.

### Class I: Lanthipeptides

**Fig. 2:**
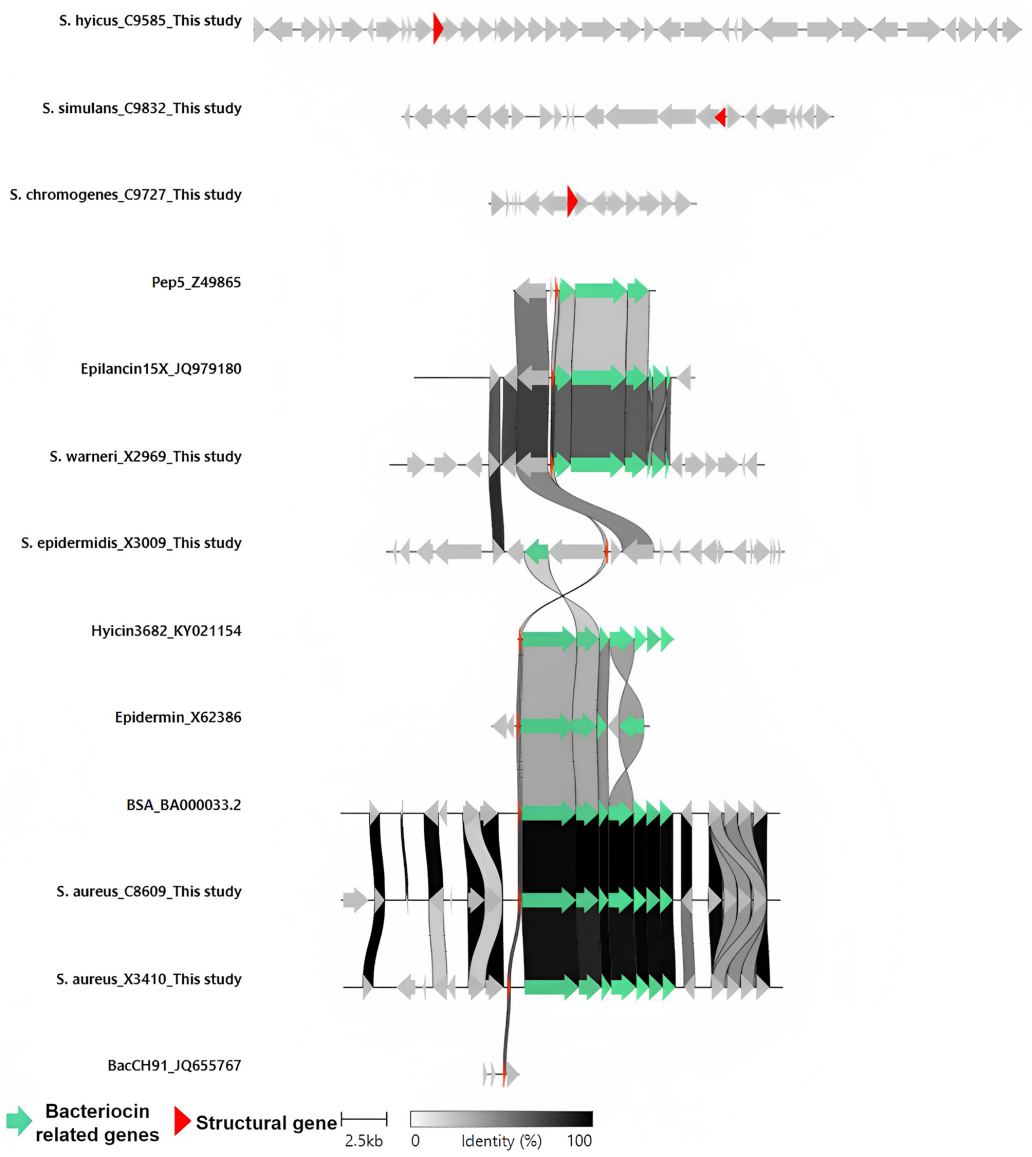
Genetic environment comparison between the bacteriocin gene clusters (BGCs) coding for lanthipeptides (class I) in the antimicrobial-producing isolates detected in this study and those selected as references from the databases. This figure is in color in the online version but in black and white in the print version

The incidence of lanthipeptides was particularly noteworthy and detected in ~32% of the evaluated bacteriocin-producing isolates (*n* = 7) (Fig. 1). A comparison between the lanthipeptide BGC operons was performed following the same criteria in all the used sequences. Figure 2 represents the genetic environment of the BGCs detected in this study (*S. aureus* X3410 and C8609, *S. hyicus* C9585, *S. warneri* X2969, *S. epidermidis* X3009, *S. simulans* C9832 and *S. chromogenes* C9727) and those previously described (accession number): bacCH91 (JQ655767), hyicin3682 (KY021154), epidermin (X62386), BSA (BA000033.2), pep5 (Z49865), and epilancin15X (JQ979180).

The *S. aureus* X3410 and C8609 BGC revealed a high degree of identity to the bacteriocin bacCH91 (JQ655767), BSA (BA000033.2), epidermin (X62386), and hyicin3682 (KY021154). On the other hand, the *S. warneri* X2969 BGC showed a close identity with the coding operon of epilancin15X (JQ979180) also related with pep5 BGC (Z49865). Regarding *S. epidermidis* X3009 BGC, slight identities were detected between the predicted structural genes but not with the rest of the encoding genes. Moreover, the *S. simulans* C9832 and *S. hyicus* C9585 lanthipeptide operons were classified separately from the rest of BGCs (Fig. 2).

Finally, the BGC predicted on the *S. chromogenes* C9727 genome was identified as hominicin. Since only the partial amino acid sequence of this bacteriocin has been reported [53], further studies will be performed to determine their identity.

**Fig. 3:**
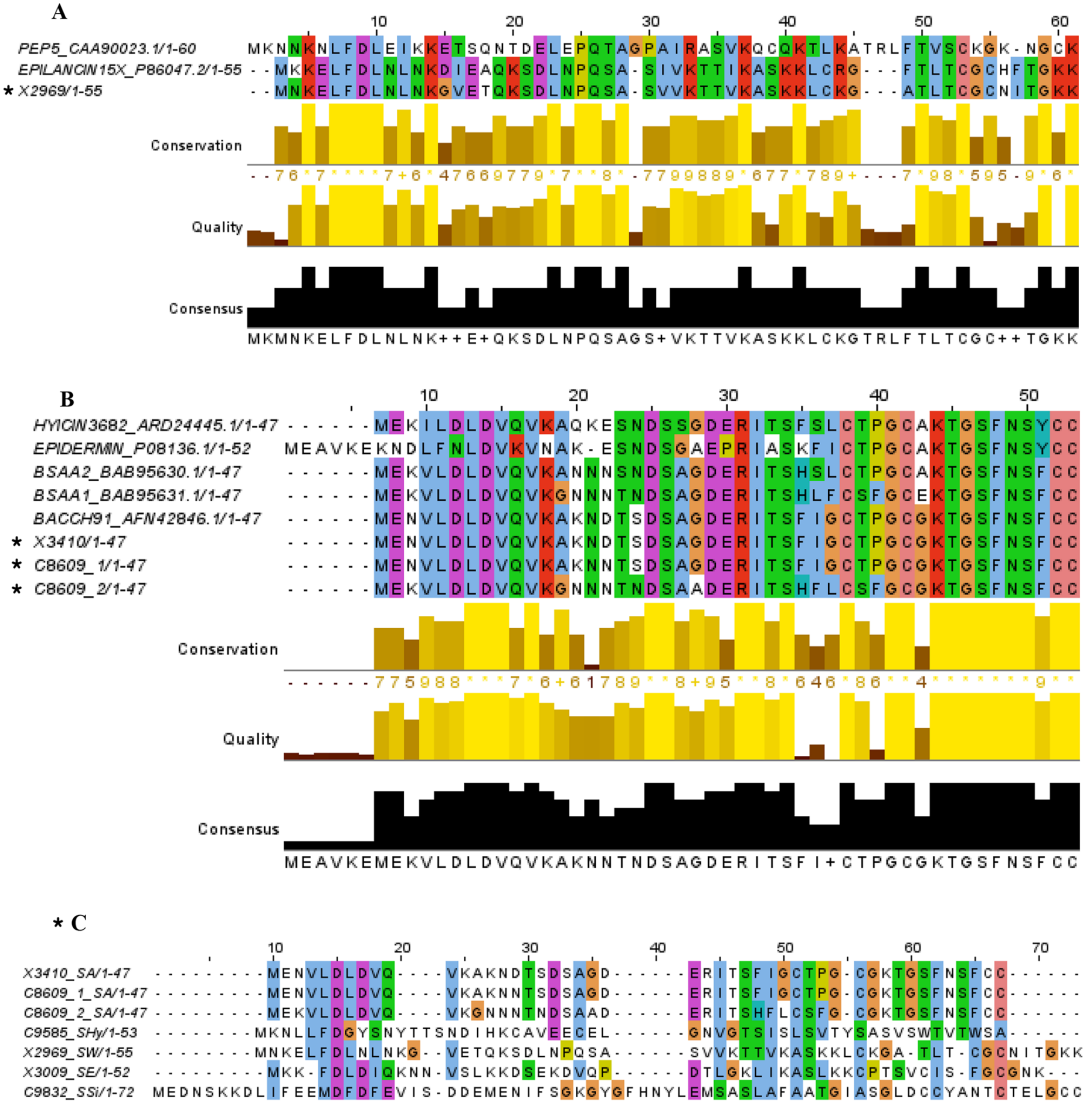
Sequence alignments of the structural peptides coding for lanthipeptide-like bacteriocins (detected in this study*) and those previously described and published in public databases used as reference. **A** *S. warneri* X2969 and the pre-peptide of epilancin15X and pep5 bacteriocins. **B** Hyicin 3682, epidermin, BsaA1, BsaA2, bacCH91 peptides with those detected in *S. aureus* C8609 isolate and *S. aureus* X3410. **C** Structural peptides detected among the genomes included in this study. * Symbol to indicate the structural peptide of the isolates included in this study. This figure is in color in the online version but in black and white in the print version

Comparative analysis presented in Fig. 3 revealed 100% amino acid identity between the bacteriocin structural peptide of *S. aureus* X3410 and C8609 and the BacCH91 structural peptide. Moreover, only slight differences were observed when comparing the bacteriocin structural pre-peptides of *S. aureus* C8609 and X3410 with the BsaA2 (83% of identity) and BsaA1 (92%) amino acid sequences. In relation to the BGC of *S. warneri* X2969, an 82% of identity was detected with the Epilancin15X structural pre-peptide. In addition, antiSMASH analysis allowed us to identify a lanthipeptide V-like BGC in the *S. hyicus* C9585 genome. Noteworthy, the BGCs of bacteriocin-producing *S. epidermidis* X3009 and *S. simulans* C9832 did not show homologues in the database, indicating the detection of two putatively new lanthipeptides (Fig. 3).

### Class II: Bacteriocins

Genome analysis also led to the identification of three types of class II bacteriocins. The BGC for lactotococcin972 was predicted in six genomes, and after genomic environment comparisons, all but one potential lactococcin972 BGCs predicted revealed identity with the structural gene of the *L. lactis*_pBL1_NC004955.1 reference sequence (Fig. S1). Moreover, high identities were observed when considering the lactococcin972 BGC predicted among the genomes included in this study, specially between *S. aureus* C8609 and C5802 and *S. sciuri* X3041 and X3011. Contrary, the BGC predicted to code for lactococcin972 in the *S. simulans* C9832 genome did not show relevant identity neither with the reference sequences considered nor with the rest of the predicted operons.

We also detected the identical structural gene of the bacSp222 bacteriocin in the genomes of the two *S. pseudintermedius* isolates recovered from the same human-pet zoonosis case. This bacteriocin was described for the first time in the plasmid p222 of *S. pseudintermedius*, used as reference (CP011490.1) [54]. Finally, a Class II type BGC was predicted in *S. xylosus* C9255 which was related to the blp bacteriocin family [55].

### Circular Bacteriocins

In the present study, three putative novel circular BGCs have been identified in *S. hyicus* C9581, *S. xylosus* C9255, and *S. chromogenes* C9838 isolates recovered from wild animals. The detected coding genes showed identity with the conserved domain of uberolysin, a circular bacteriocin firstly detected in *Streptococcus uberis* [56]. Comparative analysis of the BGCs of the potential circular bacteriocin carriers predicted in the present study and those used as references (*S. aureus*_WH39_CP060492, *S. uberis*_DQ650653 and *S. aureus* _pRJ101_Aureocyclicin4185_KF836421) was performed. A high identity was exhibited between one of the BGC predicted in the *S. chromogenes* C9838 and one of *S. xylosus* C9255 genomes with the operon coding for the circular bacteriocin described for *S. aureus* WH39. Additionally, these BGCs were closely related with the aureocyclicin 4185 BGC and the strongest identity was detected when compared with the BGC predicted in the *S. hyicus* C9581 genome. Finally, the other BGC predicted to code for a circular bacteriocin in *S. chromogens* C9838 could be considered as an unrelated genetic system (Fig. S2).

### Non-Ribosomal Peptides

Regarding NRPs, operons related with this type of secondary metabolites were predicted in six genomes from a wide variety of species and origins (Table 1). Genome analysis revealed high diversity between them, and no bacteriocins were detected except for the two clinical *S. lugdunensis* genomes (9%), carrying the lugdunin BGC. Interestingly, comparisons with the lugdunin coding operon revealed high identity > 95% with the first discovered lugdunin producer (*S. lugdunensis*_IVK28_CP063143.1) (Fig. S3) [57].

### Thiopeptides

Previous studies by our group confirmed the production of MP1 bacteriocin by environmental staphylococcal isolates using mass-spectrometry analysis [58]. Based on this finding, we decided to further characterize these isolates by genome mining tools. Thus, the BGC for MP1 thiopeptide production were detected in sequences of the four bacteriocin-producing isolates included in this study (*S. aureus* C5802 and *S. hominis* C5835 isolates from environmental samples; two *S. sciuri* X3011 and X3041 isolates from food).

**Fig. 4:**
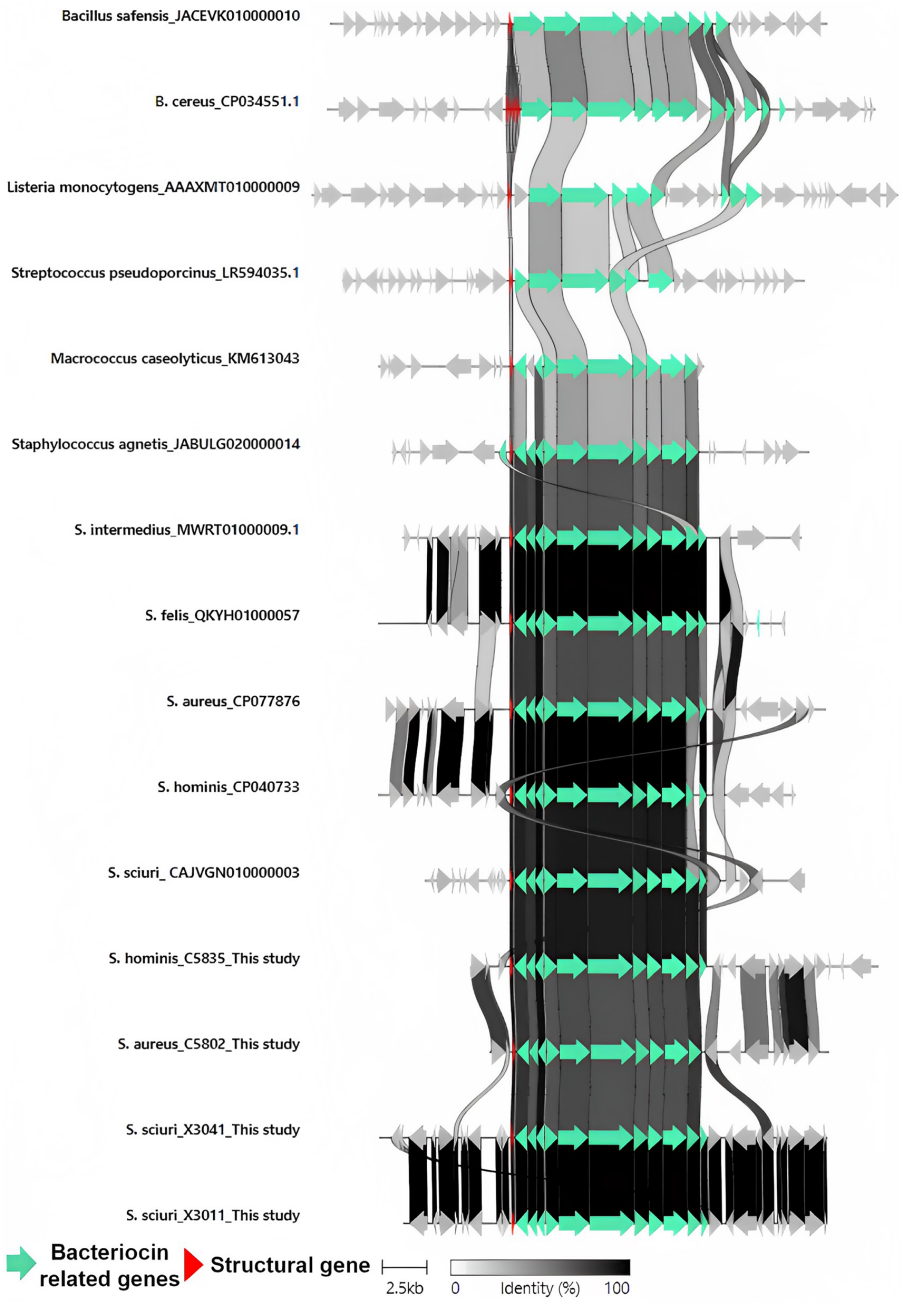
Genetic environment comparison of MP1 thiopeptide gene clusters of *Bacillus safensis* (SDG14_10_1/JACEVK010000010), *Bacillus cereus* (ATCC14579/CP034551), *Listeria monocytogenes* (FDA802499/AAAXMT010000009), *Streptococcus pseudoporcinus* (NCTC5385/LR594035), *Macrococcus caseolyticus* (pBac115/KM613043), *Staphylococcus agnetis* (4244/JABULG020000014), *S. intermedius* (14S03307/MWRT01000009), *S. felis* (F30k1271111/QKYH01000057), *S. aureus* (358/CP077876), *S. hominis* (34/CP040733), *S. sciuri* (IMDOS72p/CAJVGN010000003), *S. hominis* (C5835, this study), *S. aureus* (C5802, this study), *S. sciuri* (X3041, this study), and *S. sciuri* (X3011, this study). This figure is in color in the online version but in black and white in the print version

Genetic environment comparisons between the four MP1 producers included in this study and those previously described confirmed the high identity between the BGC structures of isolates from the genus *Staphylococcus* but with clear differences to the BGCs from other genera (Fig. 4). The reference sequences used for the MP1 operon analysis were as follows (isolate reference/accession number): *Bacillus safensis* (SDG14_10_1/JACEVK010000010), *Bacillus cereus* (ATCC14579/CP034551), *Listeria monocytogenes* (FDA802499/AAAXMT010000009), *Streptococcus pseudoporcinus* (NCTC5385/LR594035), *Macrococcus caseolyticus* (pBac115/KM613043), *Staphylococcus agnetis* (4244/JABULG020000014), *S. intermedius* (14S03307/MWRT01000009), *Staphylococcus felis* (F30k1271111/QKYH01000057), *S. aureus* (358/CP077876), *S. hominis* (34/CP040733), and *S. sciuri* (IMDOS72p/CAJVGN010000003). Moreover, close to 100% identity was observed among *S. intermedius* (14503307) and *S. felis* (F30k1271111), between *S. aureus* (358) and *S. hominis* (34), and between the two independent *S. sciuri* isolates*.* In addition, the DNA sequence analysis of the MP1 genetic environment revealed the closest relationship between the operon identified in *M. caseolyticus* (pbac115) with those found in *Staphylococcus*. The strongest differences were observed between *Staphylococcus* and *M. caseolyticus* (115) MP1 operons with those described in *Bacillus* spp. isolates (SDG14_10_1 or ATCC14579) (Fig. 4).

For deeper research of the MP1 BGC detected among the isolates included in this study, a global comparison with the NCBI database was performed. Individual BlastN analysis with each of the contigs carrying the MP1 BGC revealed a close affinity between the MP1 operon of our four carrier genomes and a complete plasmid coding for MP1 BGC of *S. hominis_*34 (CP040733.1). Moreover, the tight relationship between the entire MP1 plasmid of *S. hominis_*34 and of *S. hominis* C5835, recovered from this study, is noteworthy. In this respect, the BlastN analysis revealed a high identity and coverage percentage (> 90%), differing in 5 genes coding for ATPases or genes with unknown function. This identity was lower when comparing the MP1 BGC of the *S. aureus* C5802 isolate with *S. hominis_*34. However, high identity was observed between the *S. aureus* C5802 and *S. hominis* C5835 MP1 operons, also when comparing the flanking areas so it can be suggested that these two isolates could share a common genetic element. Finally, the presence of identical additional genes upstream of the MP1 operon in the two *S. sciuri* from this study and the selected reference (MP1 coding plasmid of *S. sciuri* IMDOS72) should be mentioned. Further analysis should be carried out to confirm the relation of the four MP1 BGC detected in this study to mobile genetic elements such as plasmids and their transmission capability.

**Fig. 5:**
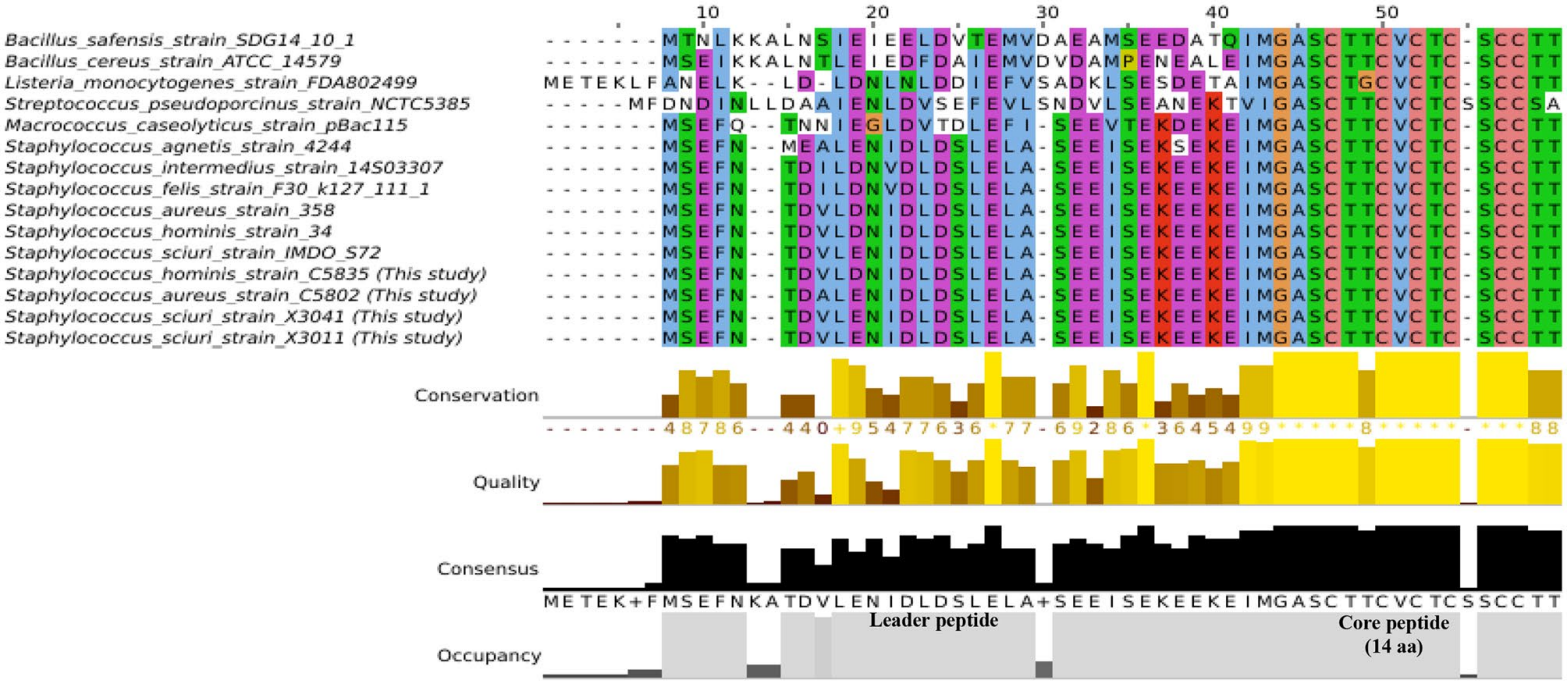
Differences at the protein level of the structural gene coding for the thiopeptide MP1: *B. safensis* (SDG14_10_1), *B. cereus* (ATCC14579), *L. monocytogenes* (FDA802499), *S. pseudoporcinus* (NCTC5385), *M. caseolyticus* (pBac115), *S. agnetis* (4244), *S. intermedius* (14S03307), *S. felis* (F30k1271111), *S. aureus* (358), *S. hominis* (34), *S. sciuri* (IMDOS72p), *S. hominis* (C5835, this study), *S. aureus* (C5802, this study), *S. sciuri* (X3041, this study), *S. sciuri* (X3011, this study). This figure is in color in the online version but in black and white in the print version

Moreover, differences in the MP1 structural gene were studied at the protein level (Fig. 5). Regarding the amino acid sequence alignment, it is important to note that *Staphylococcus* isolates were all identical when considering the MP1 structural gene, except for *S. agnetis* from the database which presented two amino acid changes in the leader peptide. Moreover, the high identity between *Staphylococcus* and *M. caseolyticus* sequences was outstanding, which only differs in a few amino acids within the leader peptide. On the other hand, the comparison with the other isolates included in this study revealed huge differences in the leader peptide sequences and even punctual amino acid exchanges and insertions within the mature peptides of *L. monocytogenes* and *S. pseudoporcinus*, likely affecting the structure and/or antimicrobial activity of their bacteriocins.

## Discussion

The new genome mining tools offer an important technological resource in the discovery of novel natural products based either on the detection of bacteriocin structural genes or other bacteriocin-associated genes [59]. In this study, ~82% of the investigated bacteriocin-producing *Staphylococcus* isolates (18 out of 22 genomes) carried at least one bacteriocin coding gene among which a large diversity was observed independently of isolate origin (human, pet, wild animals, food, and environmental). Here, five types of BGCs were identified among 18 bacteriocin-producing isolates, coding for bacteriocins of class I (lanthipepthides), class II (lactococcin972, bacSp222, and blp family bacteriocin), class IV (circular bacteriocins), NRP, and thiopeptides. Interesting relationship between related *Staphylococcus* species and the type of bacteriocin has been observed.

Focusing on lanthipeptides, they are characterized by their small (< 5 kDa) size, their post-translational modifications and they contain lanthionine or β-methyllanthionine residues in their structure [60, 61]. In the present work, seven lanthipeptide-like BGCs have been described among the 22 studied genomes, and genomic comparisons with those bacteriocin clusters previously referred in the literature allowed us to classify these putative new bacteriocins into class I. Upon lanthipeptide BGC identification among bacteriocin-producing staphylococci, bioinformatic analyses indicated the highest identity of the bacteriocins predicted in this study to the reference ones: (i) the BSA bacteriocin discovered from an MRSA strain involved in community-acquired infections [62]; (ii) the bacCH91 bacteriocin reported by Wladyka and colleagues in 2013 [63], although they only reported the structural gene; (iii) the epilancin15X antimicrobial peptide firstly detected in a clinical *S. epidermidis* isolate [64]. These results showed a close relationship between the putative structural genes detected in the newly investigated isolates and those previously reported, which suggests the detection of new variants of both BSA and epilancin15X bacteriocins. In addition, a lanthipeptide-like BGC type (V) was detected in the *S. hyicus* C9585 genome showing identity with those recently reported elsewhere [65, 66].

Interestingly, the BGCs detected in *S. warneri* and *S. epidermidis* bacteriocin-producing isolates seemed to be putative new lanthipeptide-like bacteriocins. Lanthipeptide BGC are characterized by the presence of a structural gene which is post-translationally modified by dehydratase, phosphatase, glycosidase, cyclase, oxidase enzymes, and ABC transporters [18]. Moreover, the presence of a C-terminal core region in the structural gene of *S. simulans* C9832 suggests that this operon could code for a type I lanthipeptide, while the one detected in *S. epidermidis* X3009 revealed higher identity to pep5 bacteriocin (Figs. 2 and 3). Future work will focus on the optimization of the extraction conditions to obtain inhibition in the well diffusion assay and to finally confirm the proteinaceous nature of the inhibitory compound and to decipher the peptide structure. Moreover, the use of heterologous expression systems will be important for future validation of the in silico screening studies [67–69].

Focusing on class II bacteriocins, lactococcin-like clusters were identified in six out of the 22 bacteriocin-producing isolates included. These bacteriocins are described as linear non-pediocin-like molecules. Among them, lactococcin972 produced by *Lactococcus lactis* subsp. *lactis* IPLA 972 is the most representative and frequent [70]. Figure S1 shows a lactococcin-like operon comparison, and all clusters contained the precursor of lactococcin972 domain.

In addition, the bacSp222 bacteriocin of class II has also been detected in this study. It was the first reported bacteriocin produced by *S. pseudintermedius* [54], and its genetic cluster organization revealed identity to aureocin A53. Moreover, bacsp222 is characterized by its bactericidal activity and virulence capacity that acts in modulating the immune system of the host [54].

The recently proposed class IV bacteriocins are circular peptides, formed by the post-translational covalent linkage between their carboxy and amino termini [71]. Currently, aureocyclicin 4185 from *S. aureus* 4185 is the first circular bacteriocin of *Staphylococcus* included in this category [72]. In this respect, we identified here two putative novel BGCs encoding circular bacteriocins of the circularin A/uberolysin family without identity to the previously reported circular staphylococcins (Fig. S2). Moreover, the *S. hyicus* C9581 isolate seems to carry the aureocyclicin 4185 bacteriocin coding genes.

Finally, cyclic lugdunin, which was the first NRP described among staphylococci and is considered as an independent class of antibacterials, was detected in two clinical *S. lugdunensis* isolates of the present work. Likewise, Zipperer and collaborators discovered lugdunin in a nasal *S. lugdunensis* isolate with antimicrobial activity against a wide range of Gram-positive bacteria including methicillin-resistant *S. aureus* (MRSA) [57]. The NRPS operon is about 30 kbp and encodes four genes (*lugABCD*) for the biosynthesis of lugdunin. Not all *S. lugdunensis* produce antimicrobial peptides although the lugdunin BGC seems to be conserved in this species and interestingly, the GC-content indicates horizontal transfer to *S. lugdunensis* from another bacterial species [57].

Regarding the bacteriocin classification proposed by de Freire Bastos in 2020, thiopeptides could be considered as a new class of staphylococcins (Class VI) [13]. Thiopeptides are sulfur-containing, ribosomally produced and highly post-translationally modified peptides with strong inhibitory and competitive potential [73, 74]. Most likely, MP1 (originally designated micrococcin) was firstly discovered in a strain of *Micrococcus* by its activity against *Mycobacterium tuberculosis* and it was characterized as a hydrophobic and heat-stable molecule with high activity against a wide range of Gram-positive bacteria [75]. So far, MP1 has been isolated from different genera, including *Micrococcus*, *Staphylococcus*, *Streptococcus*, and *Bacillus* spp., and origins (food, humans, and animals).

Focusing on *Staphylococcus*, MP1 production has been reported for isolates of *S. equorum* from cheese [76], *S. epidermidis* [77], *S. felis* from cats [78], *S. hominis* recovered from human skin [79], *S. sciuri* [29], and recently, *S. aureus* [30]. In the present study, MP1 production has been verified at the genetic level among four bacteriocin-producing isolates (~18%) with high antimicrobial activity against both MSSA and MRSA: two *S. aureus* and *S. hominis* isolates from environmental samples (river water) and two *S. sciuri* isolates recovered from raw meat chicken.

Comparison of the MP1 BGCs (Fig. 4) revealed major genetic differences between the staphylococcal isolates and *Bacillus*. First, the number of structural genes can differ between both genera (one for *Staphylococcus* and up to four for *Bacillus cereus*). Next, the staphylococcal strains appear to produce only one product [77], while a mixture of similar thiopeptides with different post-translational modifications [thiocillin I, II, III, MP1 and micrococcin P2 (MP2)] have been reported among *Bacillus* [80]. Only after horizontal gene transfer of the MP1 BGC into *S. aureus* RN4220, a yet uncharacterized by-product of MP1 could be detected [30]. In this study, the strong capacity of MP1 to force RN4220 to change its metabolic capacity via *citZ* mutation was highlighted. In addition, the comparison of the MP1 BGCs included in this study illustrates the high identity between the *Staphylococcus* isolates, especially when considering the same species and with *M. caseolyticus.*

Most staphylococcal BGCs appear to be associated with mobile genetic elements such as plasmids, transposons, IS-elements, or chromosomal islands [28, 30, 57]. Hence, BGCs can be transferred between strains and lineages and are important genetic determinants of competitive fitness within a given habitat [30]. Due to the great diversity of staphylococcal isolates and origins detected among our bacteriocin-producing isolates and the high frequency of MP1 carrier detection, the occurrence of certain mechanisms of BGC transfer could be assumed as mentioned above.

The analysis of the genomes included in this study allowed us to identify a wide diversity of BGCs and more concretely, the comparison of the genetic environment of MP1 revealed identity to the reference plasmids of *S. hominis* and *S. sciuri*, indicating that the BGCs are plasmid-encoded. However, although the detection of *rep* sequences by PlasmidFinder, third-generation sequencing technologies, such as PacBio [81] or Oxford Nanopore (ONT) [82] instruments are recommended to confirm the presence of transferrable BGC.

## Conclusion

In conclusion, our findings revealed a great abundance and diversity of bacteriocin gene clusters including unique systems and unfrequently detected among staphylococcal genomes. In this respect, the genus *Staphylococcus* and specially CoNS isolates have been confirmed as a valuable source of new peptide structures with promising functionalities for treatment and prevention. Moreover, the OneHealth perspective should be accentuated as a good perspective for further research on the alternatives for the AMR crisis.

## Supplementary Information

Below is the link to the electronic supplementary material.

Supplementary file1 (DOCX 400 KB)

## Data Availability

All data generated or analyzed during this study are available within this paper and its supplementary information files. References [75–79] corresponds to Supplementary material (Table S2). Illumina reads for 21 staphylococcal isolates included in this study can be found at the BioProject PRJNA974190 and C5802 is registered with ERS659514 accession number (ENA). The respective BioSample numbers are indicated in Supplementary Table S2.
