# Supplementary material for: Genomic Analysis of Bacteriocin-Producing Staphylococci: High Prevalence of Lanthipeptides and the Micrococcin P1 Biosynthetic Gene Clusters
Source: Probiotics Antimicrob Proteins. 2023 Aug 26;17(1):159–74. doi: 10.1007/s12602-023-10119-w (PMC11832629; doi:10.1007/s12602-023-10119-w)
Supplement: Supplementary file 1 — Supplementary file1 (DOCX 400 KB) [file 12602_2023_10119_MOESM1_ESM.docx]

**Supplementary material**

**Table S1.** Species, origins, and bacteriocin-producing isolates of the 1205 CoPS and CoNS isolates evaluated for antimicrobial activity in previous studies [31,32,33].

|  |  | **Number of isolates tested/ Number of bacteriocin-producing isolates** | | | | | |
| --- | --- | --- | --- | --- | --- | --- | --- |
| **Type of staphylococci** | **Species** | **Total** | **Human** | **Food** | **Wild animal** | **Pet** | **Environment** |
| **CoPS** | *S. aureus* | 193/6 | 11/0 | 72/2 | 103/3 | 0/0 | 7/1 |
|  | *S. pseudintermedius* | 60/16 | 9/1 | 1/0 | 0/0 | 50/15 | 0/0 |
|  | *S. delphini* | 31/0 | 0/0 | 19/0 | 12/0 | 0/0 | 0/0 |
|  | **Total CoPS*** | **284/22** | **20/1** | **92/2** | **115/3** | **50/15** | **7/1** |
| **CoNS** | *S. sciuri* | 352/15 | 0/0 | 23/2 | 320/13 | 0/0 | 9/0 |
|  | *S. saprophyticus* | 75/0 | 0/0 | 39/0 | 11/0 | 0/0 | 25/0 |
|  | *S. lentus* | 62/0 | 0/0 | 16/0 | 42/0 | 0/0 | 4/0 |
|  | *S. xylosus* | 56/3 | 0/0 | 7/0 | 32/3 | 0/0 | 17/0 |
|  | *S. epidermidis* | 54/5 | 4/0 | 17/4 | 21/1 | 0/0 | 12/0 |
|  | *S. fleuretti* | 29/0 | 0/0 | 14/0 | 15/0 | 0/0 | 0/0 |
|  | *S. chromogenes* | 37/10 | 0/0 | 7/3 | 28/7 | 0/0 | 2/0 |
|  | *S. warneri* | 26/6 | 1/0 | 24/6 | 0/0 | 0/0 | 1/0 |
|  | *S. vitulinus* | 24/0 | 0/0 | 5/0 | 19/0 | 0/0 | 0/0 |
|  | *S. simulans* | 29/3 | 0/0 | 16/0 | 10/3 | 0/0 | 3/0 |
|  | *S. arlettae* | 22/0 | 0/0 | 0/0 | 1/0 | 0/0 | 21/0 |
|  | *S. cohnii* | 16/0 | 0/0 | 2/0 | 2/0 | 0/0 | 12/0 |
|  | *S. equorum* | 16/0 | 0/0 | 2/0 | 13/0 | 0/0 | 1/0 |
|  | *S. pasteuri* | 9/0 | 0/0 | 8/0 | 0/0 | 0/0 | 1/0 |
|  | *S. hominis* | 15/2 | 1/0 | 3/0 | 8/1 | 0/0 | 3/1 |
|  | *S. capitis* | 7/0 | 0/0 | 1/0 | 2/0 | 0/0 | 4/0 |
|  | *S. hyicus* | 7/3 | 0/0 | 2/0 | 2/3 | 0/0 | 0/0 |
|  | *S. succinus* | 6/0 | 0/0 | 0/0 | 5/0 | 0/0 | 1/0 |
|  | *S. haemolyticus* | 8/0 | 0/0 | 0/0 | 5/0 | 0/0 | 3/0 |
|  | *S. nepalensis* | 5/0 | 0/0 | 0/0 | 5/0 | 0/0 | 5/0 |
|  | *S. kloosii* | 3/0 | 0/0 | 0/0 | 0/0 | 0/0 | 0/0 |
|  | *S. schleiferi* | 3/0 | 0/0 | 0/0 | 3/0 | 0/0 | 0/0 |
|  | *S. auricularis* | 1/0 | 0/0 | 0/0 | 0/0 | 0/0 | 1/0 |
|  | *S. felis* | 1/0 | 0/0 | 0/0 | 1/0 | 0/0 | 0/0 |
|  | *S. lugdunensis* | 57/23 | 57/23 | 0/0 | 0/0 | 0/0 | 0/0 |
|  | *S. simiae* | 1/0 | 0/0 | 0/0 | 1/0 | 0/0 | 0/0 |
|  | **Total CoNS*** | **921/70** | **63/23** | **186/15** | **547/31** | **0/0** | **125/1** |
| **Total CoPS+CoNS** | | **1205/92** | **83/24** | **278/17** | **662/34** | **50/15** | **132/2** |

|  |  |  |  |  | **Antimicrobial resistance** | | | | | | | |
| --- | --- | --- | --- | --- | --- | --- | --- | --- | --- | --- | --- | --- |
| **Isolate (ID)** | **Origin** | **Reference of Phenotypical Characteristics^1^** | **Accession number** | **Molecular typing^2^** | **Beta-lactam** | **Macrolide** | **LSA^3^** | **Tetracycline** | **Phenicol** | **Fusidic-acid** | **Fosfomycin** | **Desinfectant** |
| *S. pseudintermedius* (C4502) | Pet-Dog | UR collection | SAMN35174351 | NI | *bla*Z |  |  | *tet*(M) |  |  |  |  |
| *S. pseudintermedius* (C8189) | Human | [83] | SAMN35174352 | ST241 | *bla*Z | *erm*(B) | *aph*(3')_III, *ant*(6)-Ia |  | *cat*_pc221_ |  |  | *qac*D |
| *S. pseudintermedius* (C8478) | Pet-Dog | [83] | SAMN35174353 | ST241 | *bla*Z | *erm*(B) | *aph*(3')_III, *ant*(6)-Ia |  | *cat*_pc221_ |  |  | *qac*D |
| *S. aureus* (C5802) | Environmental-Water | [84] | ERS659514 | ST130 |  |  |  |  |  |  |  |  |
| *S. aureus* (C8609) | Wild animal-Mammal | [85] | SAMN35174354 | ST11225 |  |  |  |  |  |  |  |  |
| *S. aureus* (X3410) | Food-Chicken | [31] | SAMN35174355 | ST304 |  |  |  |  |  |  |  |  |
| *S. sciuri* (C9179) | Wild animal-Bird | [86] | SAMN35174356 | NS | *mec*A1 |  | *sal*A |  |  |  |  | *qac*D |
| *S. sciuri* (C9213) | Wild animal-Bird | [86] | SAMN35174357 | NS | *mec*A1 |  | *sal*A |  |  |  |  | *qac*D |
| *S. sciuri* (C9529) | Wild animal-Bird | [86] | SAMN35174358 | NS | *mec*A1 |  | *sal*A |  |  |  |  | *qac*D |
| *S. sciuri* (X3011) | Food-Chicken | [31] | SAMN35174359 | NS | *Bla*Z, *mec*A1 | *mph*C | *sal*A |  |  |  | *fos*D | *qac*J*, qac*D |
| *S. sciuri* (X3041) | Food-Chicken | [31] | SAMN35174360 | NS | *Bla*Z, *mec*A1 | *mph*C | *sal*A | *tet*(K) |  |  | *fos*D | *qac*J*, qac*D |
| *S. chromogenes* (C9838) | Wild animal-Mammal | [87] | SAMN35174361 | NI |  |  | *sal*A | *tet*(K) |  |  |  | *qac*D |
| *S. chromogenes* (C9727) | Wild animal-Mammal | [87] | SAMN35174362 | NI |  |  |  |  |  |  |  |  |
| *S. hyicus* (C9581) | Wild animal-Mammal | [87] | SAMN35174363 | NI | *mec*A1 |  |  |  |  |  |  | *qac*D |
| *S. hyicus* (C9585) | Wild animal-Mammal | [87] | SAMN35174364 | NI |  |  |  |  |  |  |  | *qac*D |
| *S. warneri* (X2969) | Food-Chicken | [31] | SAMN35174365 | NS |  |  |  |  |  |  |  | *qac*D*, qac*J |
| *S. epidermidis* (X3009) | Food-Chicken | [31] | SAMN35174366 | ST1025 |  | *msr*A*, mph*C |  | *tet*(K) |  | *fus*B | *fos*B | *qac*D |
| *S. xylosus* (C9255) | Wild animal-Bird | [86] | SAMN35174367 | NS |  |  |  |  |  |  |  |  |
| *S. hominis* (C5835) | Environmental-Water | [84] | SAMN35174368 | ST52/ST15 | *bla*Z | *msr*A |  | *tet*(K) |  | *fus*B |  |  |
| *S. simulans* (C9832) | Wild animal-Mammal | [87] | SAMN35174369 | NS |  |  |  | *tet*(K) |  |  |  | *qac*C*, qac*D |
| *S. lugdunensis* (C9954) | Human | [32] | SAMN35174370 | ST24 |  |  |  |  |  |  |  |  |
| *S. lugdunensis* (C9161) | Human | [32] | SAMN35174371 | ST2 |  |  |  |  |  |  |  |  |

**Table S2.** Characteristics of the 22 bacteriocin-producing *Staphylococcus* isolates included in this study including the resistome and molecular characterization by WGS analysis (Bioproject accession number PRJNA974190).

^1^References: UR: University of La Rioja.

^2^Abbreviation: NI, not identified; NE; not studied.

^3^LSA: Lincosamides, Streptogramins and Aminoglycosides; *sal*A and *mec*A1 genes are intrinsically carried by *S. sciuri* species and it was not consider as an adquired resistance mechanisms.

**Table S3.** Details of the plasmidome detected in the genomes of the 22 bacteriocin-producing isolates.

^1^Abbreviations: Nind, not indicated; (-): rep proteins not identified.

| **Isolate (ID)** | **Rep Protein** | **Plasmid similarity NCBI** | **% Identity^1^** | **Coverage^1^** |
| --- | --- | --- | --- | --- |
| *S. pseudintermedius* (C4502) | - | - | - | - |
| *S. pseudintermedius* (C8189) | rep21_Plnu8 | AM399080 | 82.21 | 579/1005 |
|  | repUS12_SAP014A | GQ900379 | 100 | 620/876 |
|  | rep7a_Pre25 | X92945 | 99.79 | 939/939 |
| *S. pseudintermedius* (C8478) | rep7a_Pre25 | X92945 | 99.79 | 939/939 |
|  | rep21RC_Psk41 | AF051917 | 89.58 | 240/950 |
|  | rep21_pLNU8 | AM399080 | 82.49, 82.21, 81.28 | 337,579,358/1005 |
|  | rep21RC_pGO1 | FM207042 | 89.58 | 240/927 |
|  | repUS12B_SAP014A | GQ900379 | 1000 | 620/876 |
| *S. aureus* (C5802) | repA_repUS23 | GQ900449 | 90.72 | 937/936 |
|  | rep2_repUS46 | GQ900449 | 98.78 | 573/573 |
|  | rep3_rep5a | GQ900405 | 89.39 | 858/861 |
| *S. aureus* (C8609) | - | - | - | - |
| *S. aureus* (X3410) | rep21_Pso385 | AM990995 | 98.86 | 1050/1050 |
|  | rep7de_Pack6 | AF093750 | 99.26 | 942/945 |
| *S. sciuri* (C9179) | rep1_rep13 | AM184099 | 92.57 | 619/924 |
|  | repA_N_rep19 | GQ900399 | 94.42 | 556/984 |
|  | repA_N_rep23 | EU366902, GU237136 | 77.94 | 408/1026 |
| *S. sciuri* (C9213) | rep1_rep13 | AM184099 | 92.41 | 619/924 |
|  | repA_N_rep23 | EU366902, GU237136 | 77.89 | 407/1026 |
| *S. sciuri* (C9529) | rep1_rep13 | AM184099 | 92.57 | 619/924 |
|  | repA_N_rep19 | GQ900399 | 94.42 | 556/984 |
|  | repA_N_rep23 | EU366902, GU237136 | 77.94 | 408/1026 |
| *S. sciuri* (X3011) | rep_trans_rep7a | U35036 | 88.85 | 942/945 |
|  | rep1_rep21 | AM990995 | 96.66 | 1047/1050 |
|  | repA_N_rep19 | FR821778 | 75.22 | 900/975 |
| *S. sciuri* (X3041) | repA_N_rep15 | AE017171 | 89.96 | 956/960 |
|  | repA_N_rep19 | FR821778 | 75.22 | 900/975 |
|  | repA_N_rep21 | AM990995 | 96.67 | 1050/1050 |
| *S. chromogenes* (C9838) | rep_trans_rep7a | SAU38656 | 100 | 539/570 |
|  | repA_N_rep24a | GQ900389 | 83.87, 93.75 | 558/989, 448/989 |
| *S. chromogenes* (C9727) | - | - | - | - |
| *S. hyicus* (C9581) | rep1_rep21 | FR714928, GQ900464 | 80.91 | 503/1005 |
|  | rep_trans_rep7a | AB037671, NC007791, SAU38656, U36910 | 100 | 197/945 |
|  | repA_N_rep19 | FR821778 | 78.22 | 450/975 |
| *S. hyicus* (C9585) | - | - | - | - |
| *S. warneri* (X2969) | repA_N_rep19c | GQ900458 | 88.57 | 980/972 |
|  | repA_N_repUS23 | GQ900449 | 93.6 | 906/936 |
|  | rep2_repUS46 | GQ900449 | 88.27 | 571/573 |
| *S. epidermidis* (X3009) | Rep1_rep21 | AF051917, FM207042, FR714928, FR821780, GQ900464, GQ900485 | Nind | Nind |
|  | repA_N_rep39 | GQ900381 | 88.52 | 897/957 |
|  | repA_N_repUS9 | AF203376 | 89.18 | 924/921 |
| *S. xylosus* (C9255) | repA_N_rep19c | CP003673 | 96.19 | 972/972 |
|  | rep1_rep21 | AM990995 | 98.86 | 1050/1050 |
| *S. hominis* (C5835) | rep1_rep13_pLNU9 | AM399082 | 98.7 | 846/846 |
|  | rep1_rep21 | FR714928, FR821780, GQ900461, GQ900464, GQ900485 | Nind | Nind |
|  | rep2_repUS46 | GQ900449 | 90.56 | 551/573 |
|  | rep3_rep5c | AF447813 | 75 | 516/858 |
|  | rep3_rep5d | AF051916 | 89.91, 93.55 | 228/636, 636/636 |
|  | repA_N_rep19b | GQ900452 | 93.05 | 734/969 |
|  | repA_N_rep20 | AP003367, GQ900453 | Nind | Nind |
|  | repA_N_rep39 | GQ900465 | 98.83 | 511/954 |
|  | repA_N_rep40 | AB125341 | 94.67 | 1257/1254 |
|  | repA_N_repUS70 | GQ900405 | 92,08 | 808/1452 |
|  | reptrans_rep7a | AB037671, AM990993, GQ900417, NC007791, SAU83488, U36910 | Nind | Nind |
| *S. simulans* (C9832) | rep1_rep13 (pLNU9) | AM399082 | 83.74 | 695/846 |
|  | rep1_rep21 (pKH12) | EU168704, GQ900461 | 81.32, 87.56 | 1001/996, 844/843 |
|  | rep_trans_rep7a | AB037671, AM990993, NC007791, SAU38656, U36910 | 100 | Nind |
| *S. lugdunensis* (C9954) | - | - | - | - |
| *S. lugdunensis* (C9161) | - | - | - | - |

**
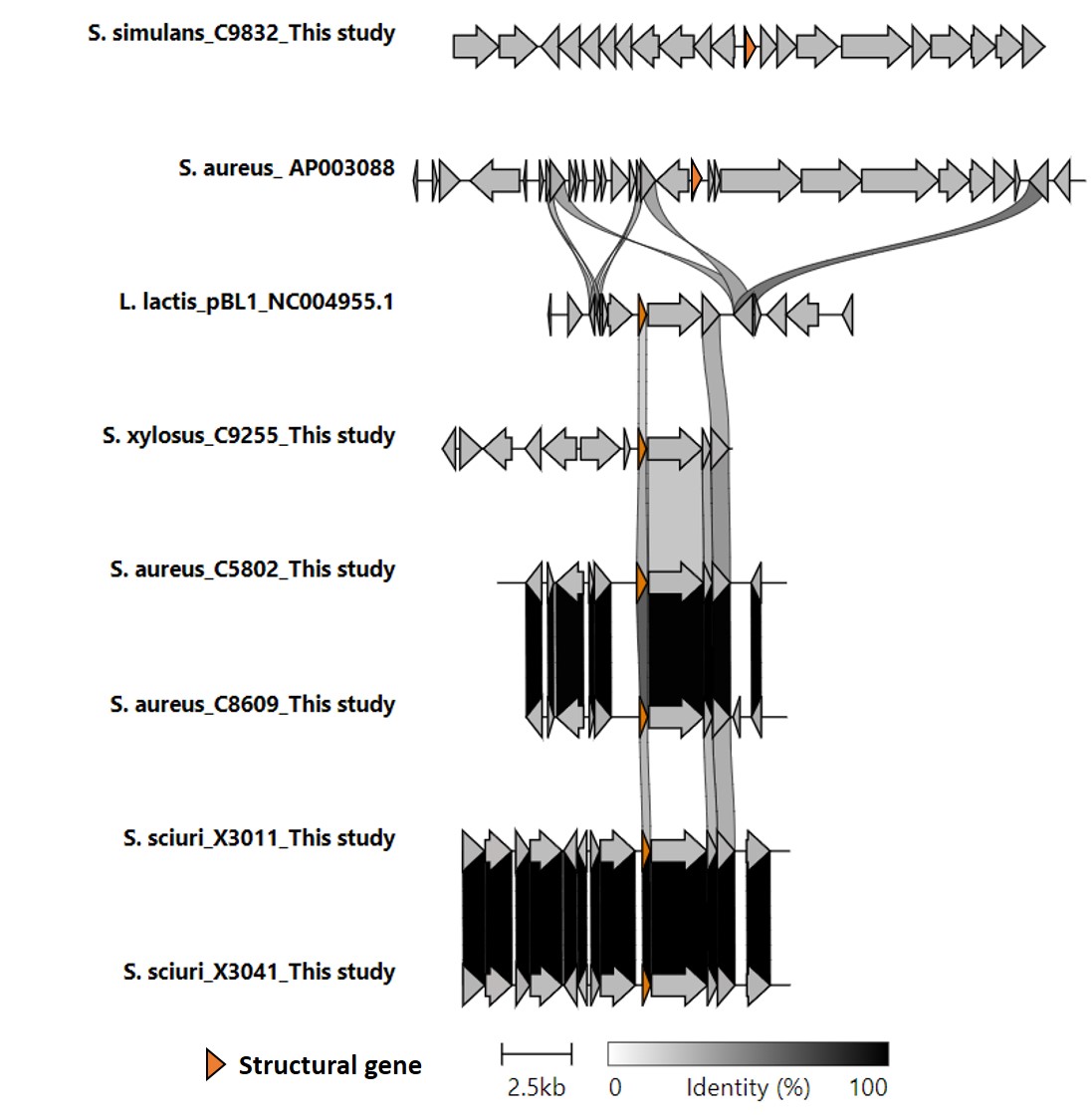
**

**Figure S1.** Genetic environment comparison between the bacteriocin gene clusters (BGCs) coding for the lactococcin972 class II bacteriocin.

*The accession numbers of the BGC coding for the bacteriocins used as reference are included in the figure.


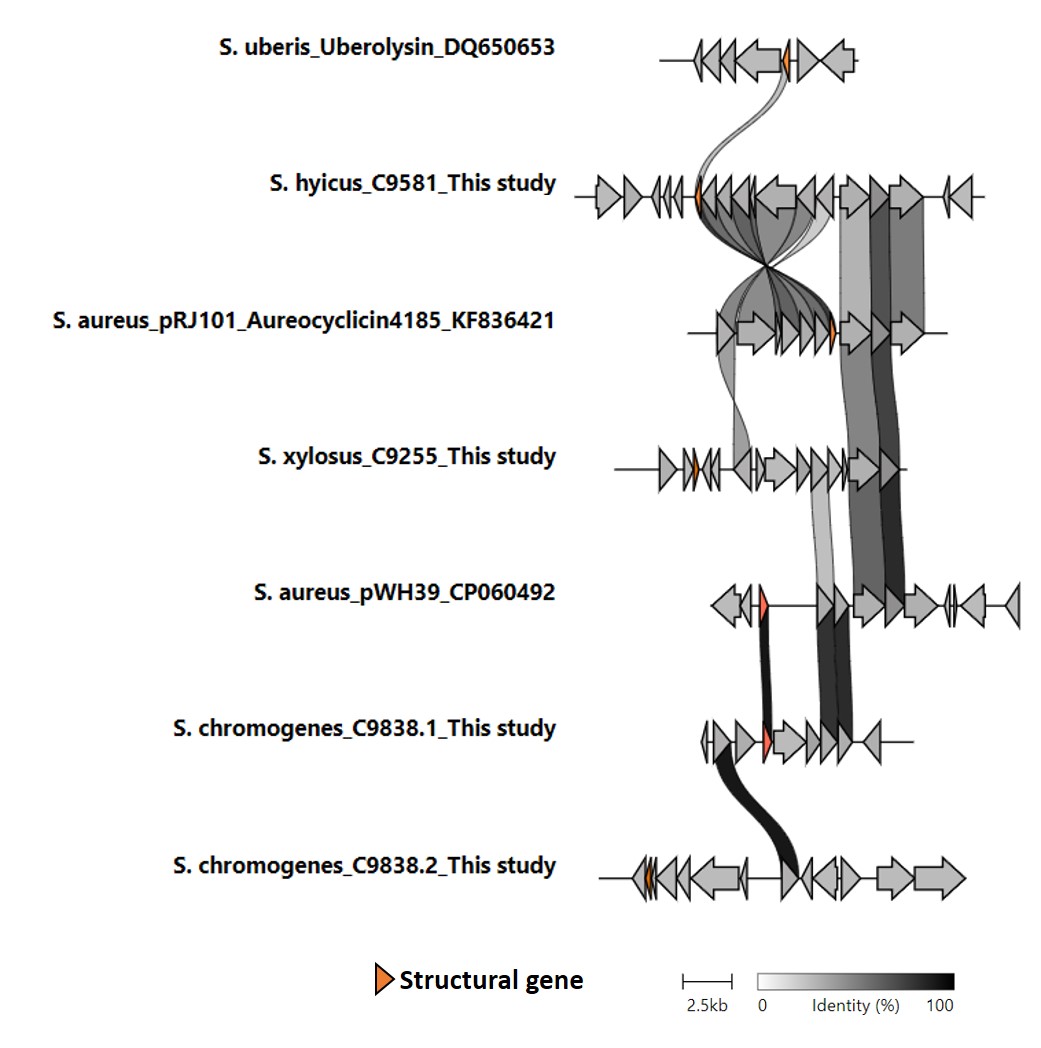


**Figure S2.** Genetic environment comparison between the bacteriocin gene clusters (BGCs) coding for the putative circular bacteriocins.

*The accession numbers of the BGC coding for the bacteriocins used as reference are included in the figure.

**
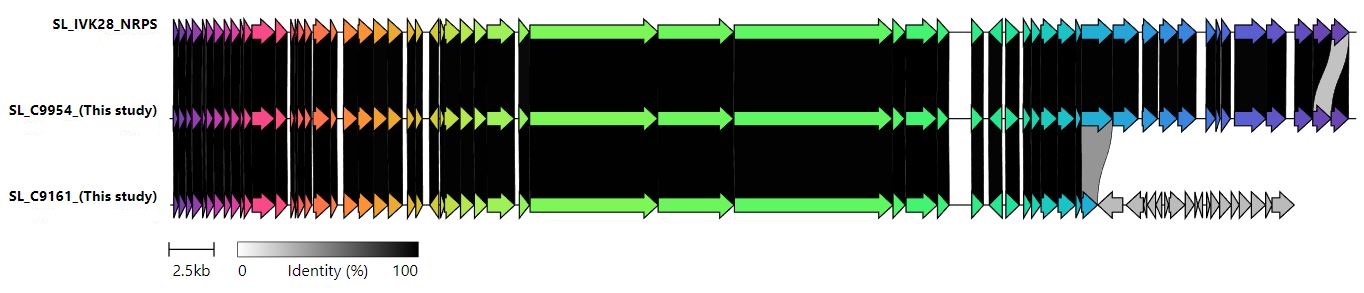
**

**Figure S3.** Genetic environment comparison between the gene clusters predicted to code for lugdunin Non-Ribosomal-Peptides (NRPs).

*The accession number for the BGC used as reference is included in the figure.
